# Convergent evolution of the arginine deiminase pathway: the ArcD and ArcE arginine/ornithine exchangers

**DOI:** 10.1002/mbo3.412

**Published:** 2016-11-01

**Authors:** Elke E. E. Noens, Juke S. Lolkema

**Affiliations:** ^1^ Molecular MicrobiologyGroningen Biomolecular Sciences and Biotechnology InstituteUniversity of GroningenGroningenThe Netherlands

**Keywords:** ADI operon, ADI pathway, ArcD, ArcE, arginine/ornithine exchange, citrulline

## Abstract

The arginine deiminase (ADI) pathway converts L‐arginine into L‐ornithine and yields 1 mol of ATP per mol of L‐arginine consumed. The L‐arginine/L‐ornithine exchanger in the pathway takes up L‐arginine and excretes L‐ornithine from the cytoplasm. Analysis of the genomes of 1281 bacterial species revealed the presence of 124 *arc* gene clusters encoding the pathway. About half of the clusters contained the gene encoding the well‐studied L‐arginine/L‐ornithine exchanger ArcD, while the other half contained a gene, termed here *arcE*, encoding a membrane protein that is not a homolog of ArcD. The *arcE* gene product of *Streptococcus pneumoniae* was shown to take up L‐arginine and L‐ornithine with affinities of 0.6 and 1 μmol/L, respectively, and to catalyze metabolic energy‐independent, electroneutral exchange. ArcE of *S. pneumoniae* could replace ArcD in the ADI pathway of *Lactococcus lactis* and provided the cells with a growth advantage. In contrast to ArcD, ArcE catalyzed translocation of the pathway intermediate L‐citrulline with high efficiency. A short version of the ADI pathway is proposed for L‐citrulline catabolism and the presence of the evolutionary unrelated *arcD* and *arcE* genes in different organisms is discussed in the context of the evolution of the ADI pathway.

## 1 Introduction

**Figure 1:**
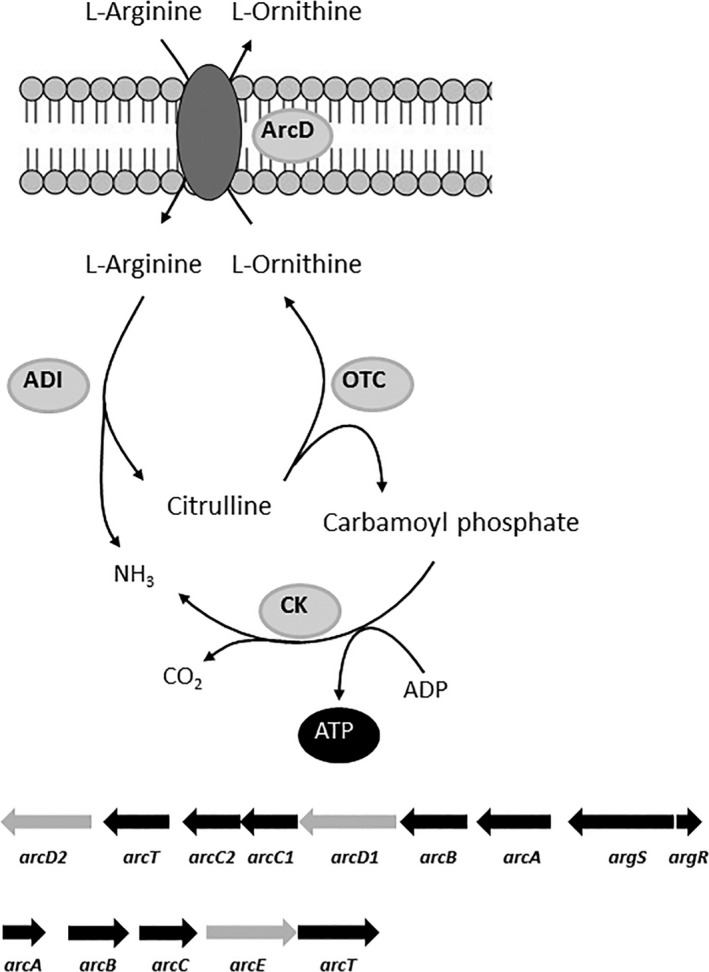
Schematic representation of the arginine deiminase (ADI) pathway. Below the scheme, the ADI gene cluster in *L. lactis* (top) and *S. pneumoniae* (bottom) are shown. ADI, arginine deiminase; OTC, catabolic ornithine transcarbamylase; CK, carbamate kinase. The *arcD1* and *arcD2* genes encode L‐ arginine/L‐ornithine exchangers (Noens et al., 2015). The *arcE* gene encodes a putative L‐arginine/L‐ornithine exchanger

The arginine deiminase (ADI) pathway is the most widespread anaerobic route for arginine degradation (Zúňiga, Pérez, & González‐Candelas, 2002). The pathway is widely distributed among bacteria and functions as a source of energy (Crow & Thomas, 1982; Cunin, Glansdorff, Pierard, & Stalon, 1986) and contributes to survival in acidic environments (Marquis, Bender, Murray, & Wong, 1987). The pathway converts L‐arginine into L‐ornithine, ammonia, and carbon dioxide with the production of 1 mol of ATP per mol of L‐arginine consumed. The conversion requires three metabolic steps (Fig. 1). First, L‐arginine is converted into L‐citrulline and ammonia, a reaction that is catalyzed by arginine deiminase (ADI, encoded by *arcA*). Subsequently, the carbamoyl moiety of L‐citrulline is transferred to phosphate by catabolic ornithine transcarbamylase (OTC, encoded by *arcB*) yielding carbamoyl phosphate and L‐ornithine. And finally, carbamoyl phosphate is used to phosphorylate ADP, yielding ATP, carbon dioxide, and ammonia, which is catalyzed by carbamate kinase (CK, encoded by *arcC*). A key player in the ADI pathway is the L‐arginine/L‐ornithine exchanger, a transporter that catalyzes stoichiometric exchange of one molecule of L‐arginine and one molecule of L‐ornithine thereby realizing concomitant uptake of the substrate L‐arginine and excretion of the end product L‐ornithine (Driessen, Poolman, Kiewiet, & Konings, 1987; Verhoogt et al., 1992). Since no metabolic energy is needed for the transport reaction, both substrates carry a single positive charge, ATP produced by the ADI pathway can be entirely used for other energy‐demanding purposes.

In bacteria, clusters containing the structural genes encoding the ADI pathway are quite diverse and may contain in addition regulatory genes, duplicated genes, and other associated genes mostly of unknown function. Moreover, the order of the genes differs among different species (Zúňiga et al., 2002). The well‐studied ADI pathway of the lactic acid bacterium *Lactococcus lactis* is encoded in a cluster of nine genes (Fig. 1). The four structural genes of the pathway are located in the center in the order *arcA‐arcB‐arcD1‐arcC1* in which *arcD1* encodes the L‐arginine/L‐ornithine exchanger. Located upstream are the putative arginyl‐tRNA synthetase gene *argS* and, transcribed in the opposite direction, the arginine repressor encoding gene *argR* (Budin‐Verneuil, Maguin, Auffray, Ehrlich, & Pichereau, 2006; Larsen, Buist, Kuipers, & Kok, 2004). Located downstream are additional copies of *arcC* and *arcD*, and *arcT*, a putative transaminase, in the order *arcC2‐arcT‐arcD2*. Recently, it was demonstrated that ArcD1 and not ArcD2 is the main L‐arginine/L‐ornithine exchanger functional in the ADI pathway. ArcD2 has been proposed to function as a putative L‐arginine/L‐alanine exchanger in a partial L‐arginine aminotransferase (ATA) pathway together with ArcT that was proposed to be an L‐arginine‐pyruvate transaminase (Noens, Kaczmarek, Żygo, & Lolkema, 2015). ArcD1 (and also ArcD2) is a secondary transporter found in the basic amino acid/polyamine transporter family (APA, Transport Classification TC 2.A.3.2 [Saier, 2000]).

The ADI cluster in the closely related species *Streptococcus pneumoniae*, both are in class lactobacillales of the Firmicutes, contain the genes encoding the metabolic enzymes ADI, OTC, and CK but no homolog of the ArcD transporter protein found in *L. lactis* is present. Instead, a gene, here referred to as *arcE*, is located downstream of *arcABC* resulting in the order *arcA‐arcB‐arcC‐arcE‐arcT* (Fig. 1). The product of the *arcE* gene is a membrane protein with no significant homology with ArcD transporters based on amino acid sequence and, accordingly, is found in a different transporter family (basic amino acid antiporter family ArcD, TC 2.A.118). Deletion of the *arcE* gene in *S. pneumoniae* resulted in reduced ADI pathway activity (Schulz et al., 2014) and arginine uptake (Gupta et al., 2013), strongly suggesting that the *arcE* gene product is the L‐arginine/L‐ornithine exchanger in *S. pneumoniae*.

Here, it is demonstrated that the *arcD* and *arcE* transporter genes are more or less equally distributed over the ADI pathways in the bacterial kingdom and the catalytic properties of the ArcE protein were determined following expression in *L. lactis*. The presence of evolutionary unrelated L‐arginine/L‐ornithine exchangers in different organisms is discussed in the context of convergent evolution at the level of the transport proteins and the pathway.

## 2 Experimental Procedures

### 2.1 Strains, media, and growth conditions

*Lactococcus lactis* JP9000 (referred to as wild type), derived from strain MG1363 and carrying the *nisRK* genes in the pseudo_10 locus (Pinto et al., 2011), is the parent of deletion mutant ∆*arcD1D2* (Noens et al., 2015). The double mutant ∆*arcD1D2* was used as host for the nisin‐inducible expression of the ArcD1 transporter of *L. lactis* JP9000 (llmg_2311, GI:500161314) (Noens et al., 2015) and the putative L‐arginine/L‐ornithine transporter ArcE of *Streptococcus pneumoniae* D39 (SPD_1978, GI:116076676) in the D1∆*arcD1D2* and E∆*arcD1D2* strains, respectively. *L. lactis* was grown at 30°C in M17 medium supplemented with 28 mmol/L glucose and in the presence of 5 μg ml^−1^ chloramphenicol and nisin at the indicated concentrations when appropriate.

Growth curves of *L. lactis* strains were recorded using a Biotek Powerwave 340 96‐well plate reader. Overnight cultures in M17 with glucose were diluted to an OD_600_ of 0.05 in 200 μl of the indicated medium and covered with 50 μl of silicon oil (1:4 of silicon oil M20 and M200) to prevent evaporation. The optical density at 600 nm was measured every 10 min for 20 hr with 30 s of shaking before each measurement.

### 2.2 Plasmid and strain construction

A 1538 bp DNA fragment harboring *arcE* was amplified by PCR from *S. pneumoniae* D39 genomic DNA using primers arcE_Spneum_F (GCGCACATGTGTGAAAAAGCTAAAAAAGGG) and arcE_Speum_R (GCGCTCT AGATTCATGGAATCACCTCACTCAC) and cloned behind the nisin‐inducible promoter P_*nisA*_ in pNZ8048 as a *Pci*I‐*Xba*I fragment (De Ruyter, Kuipers, & de Vos, 1996).

Expression strains D1∆*arcD1D2* and E∆*arcD1D2* were constructed by transformation of plasmids pNZarcD1 (Trip, Mulder, & Lolkema, 2013) and pNZarcEpn encoding ArcD1 and ArcE under control of the nisin‐inducible promoter P_nisA_, respectively, to *L. lactis* ∆*arcD1D2* using a standard electroporation protocol.

### 2.3 Transport assays

#### 2.3.1 Standard uptake in resting cells

Strains D1∆*arcD1D2* and E∆*arcD1D2* were grown in M17 with glucose to an OD_600_ of 0.5 after which expression was induced by adding 0.5 ng ml^−1^ and 0.25 ng ml^−1^ of nisin, respectively, followed by an additional 60 min of incubation for D1∆*arcD1D2* and 120 min of incubation for E∆*arcD1D2*. Cells were harvested, washed, and resuspended to an OD_600_ of 2 in ice‐cold 100 mmol/L potassium phosphate (pH 6.0) buffer containing 0.2% glucose when indicated, and kept on ice until use. An aliquot of 100 μl of cells was preincubated for 5 min at 30°C under continuous stirring, followed by the addition of [^14^C]L‐arginine, [^14^C]L‐ornithine, or [^14^C]L‐serine to final concentrations of 1.3 μmol/L, 10 μmol/L, and 6 μmol/L, respectively. Uptake was stopped by addition of 2 ml ice‐cold 0.1 mol/L LiCl and the suspension was filtered over a 0.45 μm pore size nitrocellulose filter (BA85; Schleicher & Schuell GmbH). The filter was washed once with 2 ml of ice‐cold 0.1 mol/L LiCl and submerged in Emulsifier Scintillator Plus scintillation fluid (Packard Bioscience). Radioactivity was measured by scintillation counting with a Tri‐Carb 2000CA liquid scintillation analyzer (Packard Instruments).

#### 2.3.2 Membrane potential‐driven transport

The ionophore valinomycin (Sigma) was added to a final concentration of 2 μmol/L to cells, resuspended to an OD_600_ of 10 in 100 mmol/L potassium phosphate (pH 6.0) buffer kept on ice. Cells were rapidly diluted 20‐fold to either 100 mmol/L potassium phosphate (pH 6.0) buffer or 100 mmol/L sodium phosphate (pH 6.0) buffer at 30°C containing [^14^C]L‐arginine, [^14^C]L‐ornithine, or [^14^C]L‐serine at the same concentration as above. Uptake of radiolabel was measured as described above.

#### 2.3.3 Measurement of kinetic parameters

Expression of ArcD1 and ArcE in strains D1∆*arcD1D2* and E∆*arcD1D2* was optimized for initial rate measurements by varying the nisin concentration and induction times. In case of D1∆arcD1D2, the cells were grown in M17 with glucose to an OD_600_ of 0.5, after which expression was induced by adding nisin at 0.5 ng ml^−1^ followed by an additional incubation of 30 min. In case of E∆*arcD1D2*, nisin was added at 0.5 ng ml^−1^ for 60 min (K_m_ arginine) and 30 min (K_I_ citrulline) and at 1.0 ng ml^−1^ for 60 min (K_m_ ornithine). The nisin concentration and induction time were tuned to obtain a maximal uptake of 10–15% of total radiolabel after 10 s. Initial rates were inferred from the 10 s time points. The K_m_ of ArcE for L‐arginine was determined from the initial rates of uptake measured at the concentration range of 0.16–1.30 μmol/L. The K_m_ of ArcE for L‐ornithine was determined in the range of 0.5–5 μmol/L.

### 2.4 Genome analysis

The Bacteria genome database with date stamp 12/03/2015 was downloaded from the ftp site of the National Center for Biotechnology Information (NCBI) at ftp://ftp.ncbi.nlm.nih.goc/genomes/archive/old_refseq/. A total of 166 archaeal strains and 2538 bacterial strains were extracted from the data. For each species, a single strain was picked at random resulting in a database containing 129 archaeal species and 1281 bacterial species. A formatted Blast protein database was built containing all proteins encoded in the genomes using the makeblastdb executable. Clusters of ADI proteins encoded in close vicinity on the chromosomes were identified by parallel Blast searches (Altschul et al., 1997) against the database using the encoded proteins in the cluster as queries and the blastp executable. The makeblastdb.exe and blastp.exe executables were downloaded from the NCBI ftp site. Initially, hits from different searches found on the same genome and within 12000 bp were considered to be clustered. The clustering was checked visually using a genome viewer. Multiple sequence alignments of homologous proteins were produced using Clustal‐Omega (Sievers et al., 2011).

## 3 Results

### 3.1 Distribution of *arcD* and *arcE* in bacterial *arc* gene clusters

The *arc* gene cluster found on the chromosome of *L. lactis* contains the *arcD1* and *arcD2* genes that encode L‐arginine/L‐ornithine exchangers (Noens et al., 2015; Trip et al., 2013). The *arc* cluster of *S. pneumoniae* does not contain an *arcD* homolog but instead a gene termed here *arcE*, encoding a putative L‐arginine/L‐ornithine exchanger (Gupta et al., 2013). The *arcD* and *arcE* gene products are not homologous by sequence comparison (17% sequence identity).

In a database containing the genomes of 1281 different bacterial species, a total of 124 *arc* gene clusters were identified containing at least the genes encoding the three metabolic enzymes *arcABC*, and the *arcD* and/or *arcE* gene encoding the (putative) L‐arginine/L‐ornithine exchanger (Table 1). Highest abundance was observed in the phylum Firmicutes (21%). Among the Firmicutes, the distribution is biased toward lactobacillales with almost half of the bacteria harboring the pathway (44%) while the frequency among clostridia was much lower (5%). Bacillales were in between with 21%. In the other well‐represented phylum in the database, the Proteobacteria, 10% of the members contained the *arc* cluster, again with an unbalanced distribution over the classes with 4, 9, 17, 2, and 0% for the α, β, γ, δ, and ε subdivisions, respectively. All species of the genus *Borrelia* in phylum Spirochaetales harbored the genes for the ADI pathway as did around 10% of the Actinobacteria and Tenericutes. For many of the other phyla, it is difficult to draw conclusions because they are represented with few species in the database, but it seems safe to conclude that the ADI pathway is not a trait found among Bacteroidetes and Cyanobacteria. Overall, the *arcD* and *arcE* genes were represented by the same numbers in the different clusters (63 and 64, respectively). The *arcD* gene was exclusively found in classes bacillales and clostridia of the Firmicutes and the β and δ subdivisions of the Proteobacteria, and the *arcE* gene in phyla Spirochaetales and Tenericutes. The *arc* gene clusters found in Lactobacillales and γ‐Proteobacteria, the two classes with the highest abundancy in Firmicutes and Proteobacteria, respectively, contained both genes with *arcE* slightly overrepresented over *arcD* (24/11 and 22/17, respectively). Only three clusters, all in class Lactobacillales, contained both *arcD* and *arcE*. It follows that, with few exceptions, all *arc* gene clusters contain either the *arcD* or *arcE* gene, and that both are equally abundant in the bacterial kingdom.

**Table 1:** Phylogenetic distribution of *arc* clusters encoding the metabolic enzymes ArcA, ArcB, and ArcC and the transporters ArcD and/or ArcE

Phylum	Species	Enzymes	Transporter	
		*arcABC*	*arcD*	*arcE*
*Class*				
Acidobacteria	8	–	–	–
Actinobacteria	157	10	7	3
Aquificae	9	–	–	–
Bacteroidetes	80	–	–	–
Chlamydiales	13	–	–	–
Chlorobi	10	–	–	–
Chloroflexi	12	–	–	–
Cyanobacteria	38	–	–	–
Deferribacteres	4	–	–	–
Deinococcus	16	–	–	–
Firmicutes	242	50	28	25
*Bacillales*	58	12	12	0
*Clostridia*	107	5	5	0
*Lactobacillales* a	72	32	11	24
*Other*	5	1	0	1
Fusobacteria	5	1	0	1
Nitrospirae	4	–	–	–
Proteobacteria	555	54	28	26
*Alpha* b	159	7	2	4
*Beta*	94	8	8	0
*Gamma* c	225	39	17	22
*Delta*	55	1	1	0
*Epsilon*	22	–	–	–
Planctomycetes	7	–	–	–
Spirochaetales	35	5	0	5
Synergistetes	5	–	–	–
Tenericutes	46	3	0	3
Thermotogae	15	–	–	–
Verrucomicrobia	4	–	–	–
Other	16	1	0	1
	1281^d^	124^d^	63^d^	64^d^

a Three clusters encode both ArcD and ArcE.

b Duplicate clusters in *Burkholderia xenovorans*.

c Duplicate clusters in *Pseudomonas stutzeri*.

d Total excluding entries in italics.

### 3.2 Recombinant expression *of arcE* of *S. pneumoniae* in *L. lactis*

**Figure 2:**
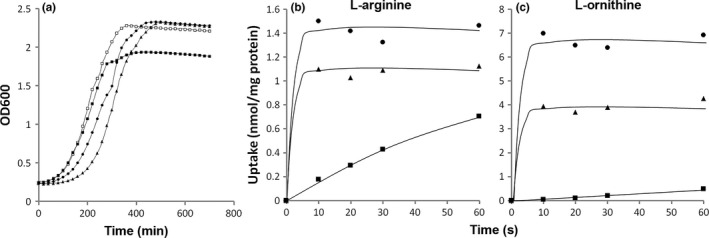
(a) Growth of wild‐type (□), Δ*arcD1D2* (■), D1Δ*arcD1D2* (●), and EΔ*arcD1D2* (▲) in M17 supplemented with glucose and 25 mmol/L L‐arginine and in the presence of 0.05 ng ml^−1^ nisin. Values are the means of at least two independent experiments. b,c. Uptake of [^14^C]L‐arginine (b) and [^14^C]L‐ornithine (c) at a concentration of 1.3 and 10 μmol/L, respectively, by resting cells of Δ*arcD1D2* (■), D1Δ*arcD1D2* (●), and EΔ*arcD1D2* (▲) grown in M17 plus glucose in the presence of 0.5 ng ml^−1^ nisin for 1 hr (D1Δ*arcD1D2*) and 0.25 ng ml^−1^ nisin for 2 hr (EΔ*arcD1D2*)

ADI pathway activity gave cells of *L. lactis* JP9000 a growth advantage resulting in a higher cell yield when cells were grown in batch in GM17 medium to which an additional 25 mmol/L‐arginine was added (Noens et al., 2015). The involvement of L‐arginine/L‐ornithine exchange in the process was evident from the lack of growth enhancement observed with the ∆*arcD1D2* strain in which the *arcD1* and *arcD2* genes were deleted and from the recovering of the growth enhancement in strain D1∆*arcD1D2* in which the *arcD1* gene is expressed from a recombinant plasmid in the deletion mutant (Fig. 2a). The gene encoding the putative L‐arginine/L‐ornithine transporter of *S. pneumoniae* D39, referred to as ArcE, was cloned in the same expression system yielding strain E∆*arcD1D2*. Growth of E∆*arcD1D2* in GM17 containing 25 mmol/L L‐arginine resulted in the same biomass yield as observed with the wild‐type and D1∆*arcD1D2* strains, indicating that ArcE can takeover the function of ArcD1 in *L. lactis* cells.

The deletion strain ∆*arcD1D2* showed severely reduced uptake activities for L‐arginine and L‐ornithine relative to the parent strain while recombinant strain D1∆*arcD1D2* showed significant and fast uptake of both substrates (Fig. 2b and c; [Noens et al., 2015]). Likewise, uptake of [^14^C]L‐arginine and [^14^C]L‐ornithine by resting cells of strain E∆*arcD1D2* containing ArcE of *S. pneumoniae* showed significant uptake of both substrates above background uptake with initial rates that were too fast to be measured under the conditions of the experiment. (Fig. 2b and c). For both L‐arginine and L‐ornithine, the steady‐state levels of uptake were higher with D1∆*arcD1D2* than E∆*arcD1D2*.

Taken together, these data support the conclusion that ArcE functions as the L‐arginine/L‐ornithine exchanger in the ADI pathway in *S. pneumoniae*.

### 3.3 Mode of transport

**Figure 3:**
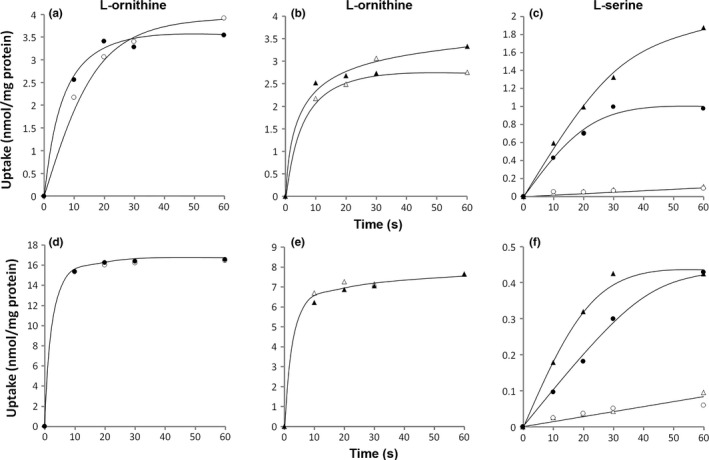
Uptake of [^14^C]L‐ornithine (a,b,d,e) and [^14^C]L‐serine (c,f) by resting cells of D1Δ*arcD1D2* (●,○) and EΔ*arcD1D2* (▲,∆). (a–c): Uptake in the presence (●, ▲) or absence (○, ∆) of 0.2% glucose. (d–f): Cells were incubated in 100 mmol/L potassium phosphate (pH 6.0) buffer in the presence of 2 uM of valinomycin and subsequently diluted 20‐fold into 100 mmol/L potassium phosphate (pH 6.0) buffer (○, ∆) or 100 mmol/L sodium phosphate (pH 6.0) buffer (●, ▲)

Previously, using a membrane vesicle system, uptake of L‐arginine and excretion of L‐ornithine by *L. lactis* was demonstrated to be a coupled process mediated by a secondary transporter catalyzing electroneutral L‐arginine/L‐ornithine exchange without the requirement of metabolic energy (Driessen et al., 1987). In agreement, uptake of L‐ornithine by whole cells of D1∆*arcD1D2* (Fig. 3a) and E∆*arcD1D2* (Fig. 3b) was not different when the cells were energized or not by the presence or absence of glucose (Fig. 3a and b). In control experiments, uptake by the same cells of L‐serine that is taken up by the pmf‐driven transporters SerP1 and SerP2 in *L. lactis* (Noens & Lolkema, 2015), was reduced to low levels when glucose was omitted from the assay mixture, (Fig. 3c) demonstrating the absence of proton motive force (pmf) in the absence of glucose. It follows that L‐ornithine uptake by D1∆*arcD1D2* and E∆*arcD1D2* is not pmf‐driven. The same experiments with L‐arginine as the substrate resulted in the same conclusion, but these experiments were hampered by the background uptake activity in the ∆*arcD1D2* deletion strain (see Fig. 2b) that was fully dependent on the presence of glucose (not shown).

Dilution of cells of *L. lactis* preincubated with the K^+^‐ionophore valinomycin in a Na^+^‐buffer generates a membrane potential due to the efflux of cytoplasmic K^+^ ions. Consequently, valinomycin treated cells of D1∆*arcD1D2* and *E∆arcD1D2* took up significant amounts of L‐serine upon 20‐fold dilution into 100 mmol/L sodium phosphate (pH 6.0) buffer, but not into 100 mmol/L potassium phosphate (pH 6.0) buffer, which is in line with an electrogenic H^+^/symport mechanism (Fig. 3f). In contrast, uptake of L‐ornithine by the same cells was similar upon dilution into 100 mmol/L potassium phosphate (pH 6.0) buffer or 100 mmol/L sodium phosphate (pH 6.0) buffer, showing that the transport catalyzed by ArcD1 and ArcE is an electroneutral event, independent of the membrane potential (Fig. 3d and e).

The results show that uptake of L‐arginine and L‐ornithine by whole cells of *L. lactis* catalyzed by ArcD1 and ArcE is consistent with an electroneutral exchange process with a cytoplasmic substrate. Most likely, the cells maintain a pool of free, positively charged amino acids like L‐arginine and/or L‐lysine in the cytoplasm that have been shown to be effective substrates for ArcD1 (Noens et al., 2015).

### 3.4 Substrate specificity of ArcE and ArcD1

Kinetic parameters of ArcD1 of *L. lactis* and ArcE of *S. pneumoniae* for uptake of L‐arginine and L‐ornithine by the D1∆*arcD1D2* and E∆*arcD1D2* strains are summarized in Table 2. Nisin inducer concentrations and induction times were adjusted to allow for initial rate measurements (see Experimental Procedures section). The affinities of ArcD1 for L‐arginine and L‐ornithine (K_m_'s of 5 and 1 μmol/L, respectively) were previously reported and added for comparison (Noens et al., 2015). Within experimental error, the affinity of ArcE for L‐ornithine was the same as observed for ArcD1 (K_m_ of 1 μmol/L), while the affinity for L‐arginine was eight times higher (K_m_ of 0.6 μmol/L). Previously, inhibition studies identified L‐lysine and L‐histidine as high‐affinity substrates of ArcD1 (Noens et al., 2015). Measuring inhibition of uptake of L‐ornithine by excess of unlabeled substrate (1 mmol/L) in E∆*arcD1D2*, indicated that ArcE has similar high affinity for L‐lysine but not for L‐histidine (not shown). Affinities of ArcE for L‐alanine and the decarboxylation products of cationic amino acids, that is, agmatine, cadaverine, histamine, and putrescine derived from L‐arginine, L‐lysine, L‐histidine, and L‐ornithine, respectively, were in the mmol/L range as was observed for ArcD1 (not shown; [Noens et al., 2015]).

**Table 2:** Kinetic parameters of the arginine/ornithine exchangers, ArcD1 and ArcE^a^

	ArcD1			ArcE		
Substrate	K_m_ (μm)	V_max_ (nmol/mg*min)	K_I_ b (μm)	K_m_ (μm)	V_max_ (nmol/mg*min)	K_I_ b (μm)
L‐Arg	5 ± 1^c^	30 ± 23^c^		0.6 ± 0.3	9 ± 4	
L‐Orn	1 ± 1^c^	45 ± 32^c^		1 ± 0.1	14 ± 6	
L‐Citr	–		529 ± 135	–		105 ± 14

a Numbers represent means plus standard deviations of at least two independent replicates.

b Inferred from the inhibition of L‐ornithine uptake at 2.5 μmol/L concentration.

c Noens et al., 2015 **.**

**Figure 4:**
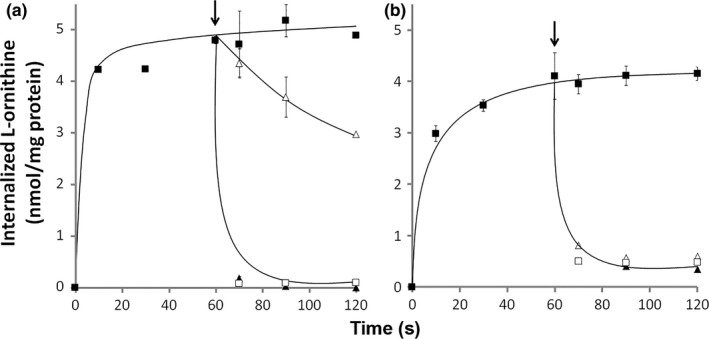
Uptake of [^14^C]L‐ornithine by resting cells of D1Δ*arcD1D2* (a) and EΔ*arcD1D2* (b) (■). At the 60‐s time point (arrow), 1 mmol/L of unlabeled L‐ arginine (▲), L‐ornithine (□), and L‐citrulline (∆) was added to the cell suspension. The initial concentration of L‐ornithine was 10 μmol/L

An interesting difference between the two transporters was observed with the ADI pathway intermediate L‐citrulline (see Fig. 1) as the substrate. Inhibition studies revealed that the affinities of ArcD1 and ArcE were two orders of magnitude lower than observed for L‐arginine and L‐ornithine. The affinity of ArcE for L‐citrulline was fivefold higher than for ArcD1 (K_I_ of 105 vs. 529 μmol/L; Table 2). Since inhibition does not necessarily reflect transport, chase experiments were performed to demonstrate the ability of the transporters to translocate L‐citrulline (Fig. 4). Cells of D1∆*arcD1D2* and E∆*arcD1D2* were allowed to take up [^14^C]L‐ornithine at 10 μmol/L initial concentration until a steady‐state level was reached (Fig. 4a and b). Addition of 100‐fold excess (1 mmol/L) of unlabeled L‐arginine and L‐ornithine resulted in rapid release of labeled L‐ornithine from the cells of both strains (see also [Noens et al., 2015]), demonstrating efficient translocation of these substrates into the cell in the exchange process catalyzed by ArcD1 and ArcE. Surprisingly, addition of 1 mmol/L L‐citrulline resulted in a very slow release of radiolabeled L‐ornithine from D1∆*arcD1D2* cells (Fig. 4a), but very rapid release from E∆*arcD1D2* (Fig. 4b). Apparently, ArcE catalyzes L‐citrulline/L‐ornithine exchange as efficient as L‐arginine/L‐ornithine and L‐ornithine/L‐ornithine exchange.

## 4 Discussion

Bacterial gene clusters encoding the ADI pathway contain either the *arcD* or the *arcE* gene that encode transporter proteins from two different transporter families (Table 1). By all accounts, ArcD and ArcE function as the L‐arginine/L‐ornithine exchangers in the pathway that take up the substrate L‐arginine and excrete the product L‐ornithine. (1) ArcD1 of *L. lactis* and ArcE of *S. pneumoniae* transport L‐arginine and L‐ornithine with high affinity (Fig. 2b and c, Table 2). (2) Both transporters catalyze efficient electroneutral exchange (Figs. 3, 4). (3) Both genes recovered the growth advantage observed with wild‐type *L. lactis* in high L‐arginine medium when expressed in the ∆*arcD1D2* deletion mutant strain (Fig. 2a). Further kinetic analysis revealed similar high affinities of the two transporters for L‐lysine and low affinities (mmol/L range) for L‐alanine and the amines agmatine, putrescine, cadaverine, and histamine—the decarboxylation products of cationic amino acids. Differences between ArcD1 and ArcE include the high affinity of ArcD1 for L‐histidine, while ArcE has low affinity, and, probably more important, the efficient L‐citrulline translocation catalyzed by ArcE and not by ArcD1. The latter property of ArcE provides additional physiological functions to the ADI pathway depicted in Fig. 1 in two ways. One, L‐citrulline/L‐ornithine exchange allows the cells to produce metabolic energy as ATP from L‐citrulline present in the medium rather than being produced in the cytoplasm from L‐arginine. L‐citrulline would be taken up from the medium in exchange with the end product L‐ornithine after which it is processed by ornithine transcarbamylase and carbamate kinase. The pathway is a short version of the ADI pathway consisting of only ArcE, ArcB, and ArcC. Two, cytoplasmic L‐citrulline as the intermediate in the ADI pathway may leave the cell by L‐arginine/L‐citrulline exchange to be taken up again in a later stage by L‐citrulline/L‐ornithine exchange. Excretion of the intermediate L‐citrulline may function as an overflow mechanism to counteract an imbalance in enzyme activity in the pathway (Liu & Liu, 1998) or as an energy uncoupling mechanism (Russel & Cook, 1995; Tempest & Neijssel, 1992).

Both physiological phenomena have been documented especially in lactic acid bacteria. Improved growth upon addition of L‐citrulline to the medium has been reported for strains of *Lactobacillus plantarum* (Arena, Saguir, & Manca de Nadra, 1999a), *Lactobacillus hilgardii*, and *Oenococcus oeni* (Arena, Saguir, & Manca de Nadra, 1999b). The strains that were shown to have an active ADI pathway as well, consumed L‐citrulline, but only in the absence of L‐arginine. The gene products responsible for the activities were not known, but the present data suggest the involvement of ArcE in the uptake of L‐citrulline. The two orders of magnitude, higher affinity of ArcE for L‐arginine compared to L‐citrulline would explain the lack of uptake of the latter in the presence of the former. It seems plausible that some lactic acid bacteria support an L‐citrulline catabolic route that is a shortened version of the ADI pathway with a ArcE homolog catalyzing L‐citrulline/L‐ornithine exchange. Several studies report the excretion of L‐citrulline from L‐arginine by cells that possess an active ADI pathway, for instance in *Mycoplasma hominis* (Schimke & Barile, 1963), *Pseudomonas putida* (Kakimoto, Shibatani, Nishimura, & Chibata, 1971; Yamamoto, Sato, Tosa, & Chibata, 1974), *Lactobacillus sakei* (Montel & Champonier, 1987), *Lactobacillus plantaris* (Jonsson, Clausen, & Raa, 1983), and *Streptococcus faecalis* (Simon, Wargnies, & Stalon, 1982). The cells possess an active ADI pathway, suggesting the involvement of L‐arginine/L‐citrulline exchange by ArcE in the excretion process. Some species, like *Lactobacillus buchneri* CUC‐3 (Liu, Pritchard, Hardman, & Liu, 1996; Liu, Pritchard, Hardman, & Pilone, 1994) and *Lactobacillus sakei* (Rimaux et al., 2013) were shown to reuptake excreted L‐citrulline after the exhaustion of L‐arginine, most likely, following the L‐citrulline catabolic pathway described above. The *arc* operon of *L. sakei* contains seven genes, *arcA, arcB, arcC, arcT, arcD, arcR* and a gene termed PTP (Rimaux et al., 2013). Interestingly, the PTP gene is a homolog of *arcE* and, therefore, the cluster is one of the few clusters that contain both *arcD* and *arcE*. It was demonstrated that deletion of PTP of *L. sakei* prevented the reuptake of L‐citrulline from the medium (Rimaux et al., 2013). The much higher affinity of ArcE for L‐arginine than for L‐citrulline is likely to result in the full conversion of L‐arginine into external L‐citrulline, before the latter is taken up again.

In many genomes, the gene encoding ArcE is annotated as *arcD*, probably because it is the only membrane protein encoded in the *arc* cluster in the absence of a true *arcD* homolog. This is unfortunate as it obscures the lack of evolutionary relationship between the *arcD* and *arcE* genes. (The same is true for the *arcT* genes in Fig. 1 that encode proteins with different functions in *L. lactis* and *S. pneumoniae*). The *arcD* gene product is a member of the APA family of secondary transporters in the Transporter Classification system (TC 2.A.3.2; [Saier, 2000;]) which is part of the Amino acid/Polyamine/Organocation (APC) transporter superfamily (Jack, Paulsen, & Saier, 2000). In the TC system, the *arcE* gene product is a member of the ArcD family (TC 2.A.118) which, again, is a very unfortunate name as *arcE* is not a member of the *arcD* gene family. The ArcD family (TC 2.A.118) is part of a different superfamily, the Ion Transporter (IT) superfamily (Prakash, Cooper, Singhi, & Saier, 2003) emphasizing the fast evolutionary distance between the *arcD* and *arcE* gene families. The MemGen structural classification of membrane proteins (Lolkema & Slotboom, 1998; Lolkema & Slotboom, 2003; Lolkema & Slotboom, 2008) takes the next step in evolutionary distance by comparing hydropathy profiles of families of proteins. The profiles represent the fold of the proteins that, in general, is better conserved in evolution than sequence. It allows for classification of transporters that do not share any significant sequence identity into the same or different structural classes. In the MemGen classification system, ArcD transporters are in the [st201]APA family in structural class ST2. The three‐dimensional structure of a number of transporters from different families in class ST2 have been resolved and they all show the so‐called “LeuT” fold (Ehrnstorfer, Geertsma, Pardon, Steyaert, & Dutzler, 2014; Fang et al., 2009; Ma et al., 2012; Malinauskaite et al., 2014; Ressl, Terwisscha van Scheltinga, Vonrhein, Ott, & Ziegler, 2009; Shaffer, Goehring, Shankaranarayanan, & Gouaux, 2009; Watanabe et al., 2010; Weyand et al., 2008; Yamashita, Singh, Kawate, Jin, & Gouaux, 2005). The ArcE putative transporters are in the [st313]ITB family (formerly known as [st313]AITC) in structural class ST3. Crystal structures of transporters from ST3 families reveal a fold that is different from the “LeuT” fold (Bolla et al., 2015; Mancusso, Gregorio, Liu, & Wang, 2012; Su et al., 2015; Wöhlert, Grötzinger, Kühlbrandt, & Yildiz, 2015). Most likely, the *arcD* and *arcE* genes do not share a common ancestor and the L‐arginine/L‐ornithine exchange activity of both is the result of convergent evolution. In the archaeal domain, the euryarchaeon, *Halobacterium salinarum* R1, and the *Halobacterium sp*. NRC‐1 contain plasmids that harbor the metabolic ADI pathway enzymes ArcA, ArcB, and ArcC clustered with a transporter protein of the sodium proton antiporter family NhaC found in MemGen structural class ST3. In *Natronobacterium gregoryi* SP1, another archaeon, a homolog of the transporter is found 4 kb downstream of the *arcABC* cluster. The transporter of *Halobacterium salinarum* was demonstrated to be the L‐arginine/L‐ornithine exchanger in the ADI pathway (Wimmer, Oberwinkler, Bisle, Tittor, & Oesterhelt, 2008). The NhaC transporter shows no significant sequence identity with the ArcD and ArcE proteins and may provide another example of a transporter that functionally converged to L‐arginine/L‐ornithine exchange activity.

The physiological advantage that drives clustering of genes on the chromosome during evolution is believed to be efficient coregulation of expression of functionally related genes. The mechanism of clustering though is still an open question for which many theories have been presented (see f.i. [Fondi, Emiliani, & Fani, 2009; Martin & McInerney, 2009]). The ADI pathway gene clusters presented in Table 1 differ in composition and gene order (not shown). The pathway requires at least the genes encoding the three metabolic enzymes, arginine deiminase, ornithine carbamylase, and carbamate kinase, and the L‐arginine/L‐ornithine exchanger. Irrespective of the diversity, the arginine deiminase encoding genes in all clusters are homologous proteins belonging to one family (*arcA*). The same is true for the OCT and CK encoding genes (*arcB* and *arcC*, respectively), but, as demonstrated here, not for the L‐arginine/L‐ornithine exchanger that belong to two different gene families in bacteria (*arcD* and *arcE*). Analysis of the gene order in the 124 bacterial clusters (Table 1) showed that the order of transcription, with few exceptions, was a*rcA* followed by *arcB* and then *arcC*. In the different clusters, the *arcD* or *arcE* gene (and other *arc* genes) are added to this sequence or inserted in between (not shown). These observations are consistent with an evolutionary model for the formation of the *arc* clusters that starts with a primordial operon *arcABC* that would encode a pathway that breaks down cytoplasmic L‐arginine to yield ATP. Cytoplasmic L‐arginine may be derived from peptidase activity following peptide uptake, a trait of many, especially lactic acid bacteria. The transporter was added at a later stage to the cluster to allow external L‐arginine to be the substrate of the new pathway encoded in the cluster. This event must have happened a number of times independently of each other resulting in different positions of the newly acquired gene and, also, in unrelated transporter genes. Transporter genes were recruited that merely could do the job of L‐arginine/L‐ornithine exchange. The model is supported by phylogenetic analysis of the genes that showed evolution of a particular cluster, characterized by the presence of *arcD* or *arcE* and gene order, as a single entity, that is, the *arcA*,* arcB*, and *arcC* genes were on the same branch in the phylogenetic tree (not shown). In this model, the formation of the ADI gene clusters is a combination of divergent and convergent evolution. Evolution of the *arcA*,* arcB*, and *arcC* genes is divergent following the evolution of species, while evolution to the final activity is convergent by the addition in multiple, independent events of transporters that are not evolutionary related but catalyze L‐arginine/L‐ornithine exchange.

## Conflict of Interest

None declared.
